# Life Expectancy Gaps Among Black and White Persons and Contributing Causes of Death in 3 Large US Cities, 2018-2019

**DOI:** 10.1001/jamanetworkopen.2023.3146

**Published:** 2023-03-10

**Authors:** Pamela T. Roesch, Nazia S. Saiyed, Emily Laflamme, Fernando G. De Maio, Maureen R. Benjamins

**Affiliations:** 1 Sinai Urban Health Institute, Chicago, Illinois; 2 Center for Neighborhood Technology, Chicago, Illinois; 3 American Medical Association, Chicago, Illinois; 4 DePaul University, Chicago, Illinois

## Abstract

**Importance:**

US cities have substantial, but varying, levels of racial mortality inequities, a consequence of structural racism. As committed partners increasingly pledge to eliminate health inequities, local data are required to focus and unify efforts.

**Objective:**

To analyze the contributions of 26 cause-of-death categories to Black to White life expectancy gaps within 3 large US cities.

**Design, Setting, and Participants:**

In this cross-sectional study, data were extracted from the 2018 and 2019 National Vital Statistics System Multiple Cause of Death Restricted Use data files for deaths by race, ethnicity, sex, age, place of residence, and underlying and contributing causes of death in Baltimore, Maryland; Houston, Texas; and Los Angeles, California. Life expectancy at birth was calculated for non-Hispanic Black and non-Hispanic White populations overall and by sex using abridged life tables with 5-year age intervals. Data analysis was performed from February to May 2022.

**Main Outcomes and Measures:**

Using the Arriaga method, the proportion of the Black to White life expectancy gap was calculated overall and by sex for each city that was attributable to 26 cause-of-death categories defined using the *International Statistical Classification of Diseases and Related Health Problems, Tenth Revision* codes for underlying and contributing causes of death.

**Results:**

A total of 66 321 death records from 2018 to 2019 were analyzed, with 29 057 individuals (44%) were identified as Black, 34 745 (52%) as male, and 46 128 (70%) as aged 65 years and older. Black to White life expectancy gaps were 7.60 years for Baltimore, 8.06 years for Houston, and 9.57 years for Los Angeles. Circulatory diseases, cancer, injuries, and diabetes and endocrine disorders were top contributors to the gaps, although the order and magnitude varied by city. The contribution of circulatory diseases was 11.3 percentage points higher in Los Angeles than in Baltimore (3.76 years [39.3%] vs 2.12 years [28.0%]). The contribution of injuries to Baltimore’s racial gap (2.22 years [29.3%]) was twice as large as in Houston (1.11 years [13.8%]) and Los Angeles (1.36 years [14.2%]).

**Conclusions and Relevance:**

By assessing the composition of Black to White life expectancy gaps for 3 large US cities and categorizing deaths at a more granular level than past studies, this study provides insight into the differing underpinnings of urban inequities. This type of local data can support local resource allocation that more effectively addresses racial inequities.

## Key Points

**Question:**

What causes of death contribute to the Black to White life expectancy gaps within 3 US cities, and how do causes differ across locations?

**Findings:**

This cross-sectional study of 66 321 death records calculated the proportion of the Black to White life expectancy gap that was attributable to 26 cause-of-death categories overall and by sex for Baltimore, Maryland; Houston, Texas; and Los Angeles, California. Circulatory diseases, cancer, injuries, and diabetes were the top contributors, although the order and magnitude varied among cities.

**Meaning:**

These findings highlight the underpinnings of urban inequities and the value of local data for developing city-specific approaches to achieve racial health equity.

## Teaser

This cross-sectional study analyzes the contributions of 26 cause-of-death categories to Black to White life expectancy gaps within 3 large US cities: Baltimore, Maryland; Houston, Texas; and Los Angeles, California.

## Introduction

The largest US cities have substantial, but varying, levels of racial mortality inequities associated with structural racism. Not only does the extent of inequity differ across cities, but city-level progress toward equity is uneven.^1,2^ As an increasing number of committed partners (eg, local policy makers, health departments, community organizations, health care systems, funders, and advocacy partners) pledge to eliminate health inequities, city-level analyses of the causes of death most implicated are needed to help focus and unify local efforts.

Data on overall life expectancy gaps between racialized groups is valuable, giving a sense of the magnitude of structural racism and how it operates from city to city. However, by itself, such analysis is limited in the extent it can guide actions and possible solutions. Identifying the precise causes of death that contribute to the non-Hispanic Black to non-Hispanic White (hereafter referred to as Black and White; the term “Hispanic” is used here because it is the term used in our data source) life expectancy gaps helps narrow the list of potential initiatives to advance equity. For example, the local health department of Chicago, Illinois, and a large local collaborative,^3^ both dedicated to addressing the city’s racial life expectancy gap, prioritized their efforts by using a decomposition analysis of Chicago’s Black to White inequity in life expectancy that identified heart disease and homicide as primary contributors.^4^

The contributions of different causes of death to the Black to White life expectancy gap (and changes in the gap over time) have been well studied at the national level. Kochanek et al^5^ found that the 3.8-year racial gap in life expectancy in the US in 2010 was primarily associated with deaths from heart disease, cancer, homicide, diabetes, and perinatal conditions. Other analyses show similar contributions by cause.^6,7^ The causes of death largely overlap across sexes, although homicide, HIV, and drug-related deaths tend to have a greater impact for male individuals, whereas chronic conditions have more impact for female individuals.^5,8^ Additional studies^9,10,11,12^ have found state-level differences in the causes of death that contribute to racial inequities, both overall and by sex.

These national-level and state-level data provide important context for more local analyses, which are critical for guiding local policies, informing funding allocations, and motivating grassroots efforts. Cities are important to focus on because 80% of US residents live in urban areas.^13,14^

Despite the potential value of local data, we found assessments on causal contributors to racial life expectancy inequities for only 2 cities (Washington, DC; and Chicago) and 1 county (Los Angeles County, California).^4,15,16^ Similar to the national studies, these studies identified chronic disease and cancer overall, and homicide among male individuals, as major contributors to racial inequities.^4,15,16^ Importantly, however, each analysis also cited other contributors with unique local relevance, such as infant mortality,^4^ HIV,^4,15^ and opioid overdose.^4^ Although these studies reveal the importance of local data in guiding strategic response to racial mortality inequities,^17^ differences in years studied and methodological approaches make even these few cases difficult to compare.

To address these gaps and to build a better understanding of variation in inequities at the city level, this cross-sectional study provides a decomposition of Black to White life expectancy gaps for 3 geographically and demographically diverse US cities: Los Angeles, California; Houston, Texas; and Baltimore, Maryland. Our results provide a snapshot of the similarities and differences across US cities, which, in turn, support the policy and program implication that to effectively address racial health inequities, efforts should be tailored to the histories and contexts of specific places.

## Methods

### Study Populations

In this cross-sectional study, we conducted a decomposition analysis of Black to White inequities in life expectancy for 3 of the 30 most populous cities in the US: Los Angeles, Houston, and Baltimore. To select these cities, we used city-level data on the size of the Black to White life expectancy gap, changes in the Black to White life expectancy gap over the past decade, and the percentage of the population identifying as Black (data not shown) to identify a demographically diverse sample with sufficient size to apply our decomposition methods. We also considered geographic variation. We excluded cities for which recent similar analyses are available (eg, Chicago and Washington, DC).^4,16^ This study was reviewed by the Mount Sinai Hospital (Chicago, Illinois) institutional review board and did not require full review or informed consent because it used deidentified data, in accordance with 45 CFR §46. Our reporting follows the Strengthening the Reporting of Observational Studies in Epidemiology (STROBE) reporting guidelines.

### Data Sources

For our decomposition analysis, we used 2018 and 2019 Multiple Cause of Death Restricted Use data files from the National Vital Statistics System to extract deaths by race, sex, age, place of residence, underlying cause of death, and contributing causes of death.^18^ Death certificate data were completed by proxy (eg, funeral director or attending physician).^19^ We categorized deaths into 5-year age intervals (<1 year, 1-4 years, 5-9 years, ..., 80-84 years, and ≥85 years). Death records with unstated age or where race was identified as anything other than non-Hispanic Black or non-Hispanic White were excluded. We included records where the place of residence, as identified by the city’s Federal Information Processing Standard (FIPS) code and state of residence, included the cities of Los Angeles, California (FIPS code 44000); Houston, Texas (FIPS code 35000); and Baltimore, Maryland (FIPS code 4000). We gathered age-specific, sex-specific, race-specific, and ethnicity-specific population data for each city from the US Census Bureau’s 2010 Decennial Census.^20^ All US Census data are self-reported. To contextually understand the demographic variation across cities, we gathered population and socioeconomic characteristics using self-reported 2019 American Community Survey 5-year estimates^21^ and 2019 data from the City Health Dashboard^22^ (Table 1).

**Table 1.** Characteristics of 3 Large US Cities

Characteristic	Los Angeles, California	Houston, Texas	Baltimore, Maryland	US overall
Mortality data^a^				
Total non-Hispanic Black deaths in 2018-2019, No.	8612	11 338	9107	692 122
Deaths of male individuals, No. (%)	4457 (51.8)	5902 (52.1)	4926 (54.1)	361 223 (52.2)
Deaths of individuals aged ≥65 y, No. (%)	5557 (64.5)	6519 (57.5)	4972 (54.6)	410 015 (59.2)
Total non-Hispanic White deaths in 2018-2019, No.	20 522	13 325	3417	4 377 817
Deaths of male individuals, No.	10 740 (52.3)	6909 (51.8)	1811 (53.0)	2 230 426 (50.9)
Deaths of individuals aged ≥65 y, No.	16 714 (81.4)	10 179 (76.4)	2277 (66.6)	3 411 608 (77.9)
Life expectancy, y				
Non-Hispanic Black	71.98	68.75	70.14	74.7^b^
Non-Hispanic White	81.55	76.82	77.73	78.8^b^
Total Black to White life expectancy gap, y	9.57	8.06	7.60	4.1^b^
Population characteristics^c^				
Total population, No.	3 966 936	2 310 432	609 032	324 697 795
Non-Hispanic Black, No. (%)	341 750 (8.6)	510 455 (22.1)	376 203 (61.8)	39 977 554 (12.3)
Non-Hispanic White, No. (%)	1 129 956 (28.5)	564 044 (24.4)	167 430 (27.5)	197 100 373 (60.7)
Children (aged 0-17 y)	821 416 (20.7)	579 554 (25.1)	125 823 (20.7)	73 429 392 (22.6)
Seniors (aged ≥65 y)	491 598 (12.4)	243 136 (10.5)	82 721 (13.6)	50 783 796 (15.6)
Socioeconomic characteristics, No. (%)				
Individuals living at or below 100% federal poverty level^c^	700 951 (18.0)	456 815 (20.1)	124 436 (21.2)	42 510 843 (13.4)
Unemployment rate^c^	136 312 (6.3)	70 465 (5.9)	25 391 (8.3)	8 713 400 (5.3)
High school completion, equivalent, or higher^c^	2 120 879 (77.5)	1 186 774 (78.9)	360 896 (85.2)	194 149 815 (88.0)
Uninsured rate^c^	451 091 (11.4)	529 920 (23.1)	39 536 (6.6)	28 248 613 (8.8)
Neighborhood racial and ethnic segregation score^d^^,^^e^	26.4	23.0	32.2	10.8
Income inequality score^d^^,^^f^	−9.1	−15.6	−21.2	−9.0

^a^ Calculated using 2018 and 2019 Multiple Cause of Death data files from the National Vital Statistics System.^18^

^b^ US mortality data were obtained from Xu et al.^20^ Data are reported to 1 decimal place.

^c^ Data are 2019 5-year estimates from the American Community Survey.^21^

^d^ Data for 2019 were accessed from the City Health Dashboard.^22^

^e^ Higher estimates indicate greater segregation.

^f^ Measured using the Index of Concentration at the Extremes, which compares households with income at the extremes of the national income distribution (the top 20% or bottom 20%) within a given location. A score of −100 indicates that all households are in the bottom 20%, 100 indicates that all households are in the top 20%, and 0 indicates greater equality. In 2019, the average of cities on the City Health Dashboard was −9.

### Measures

Using methods outlined by Chiang,^23^ we calculated life expectancy at birth for the Black and White populations overall and among male and female individuals for all 3 cities using abridged life tables with 5-year age intervals. If an age category had 0 deaths, 0.5 was used to enable the life tables and decomposition analysis to run mathematically. Leveraging approaches defined in previous studies^8,9,11,12,16^ and the *International Statistical Classification of Diseases and Related Health Problems, Tenth Revision (ICD-10)* hierarchy of codes, we categorized the underlying causes of death into 26 categories that were grouped into 13 higher-level classifications (see eTable 1 in Supplement 1 for complete list). If the underlying cause of death was categorized as an overdose and had a contributing cause of death that indicated opioid use (*ICD-10* codes T40.0-T40.4 and T40.6), the death was categorized as an opioid-related death.

### Statistical Analysis

Data analysis was performed from February to May 2022. We decomposed the Black to White life expectancy gap within each city by underlying cause of death using the well-established Arriaga method,^24^ conducting overall and sex-specific analyses. Specifically, we report the proportion of the Black to White life expectancy gap within each city that was attributable to the 26 cause-of-death categories both overall and by sex. Arriaga’s method assumes that the total contribution of a cause of death to the life expectancy gap will equal the sum of each age-specific contribution of that cause of death across age groups. Owing to these methodological underpinnings, causes of death that explain racial inequities in younger age categories (eg, homicide) contribute more to the life expectancy gap because of the number of potential years of life lost. Furthermore, when aggregated together, the different contributions of the categorized causes of death to the overall life expectancy gap will equal the total life expectancy gap in years. Specific decomposition formulas are described in detail by Preston et al^25^ and are included in the eAppendix in Supplement 1. Aligned with our study’s aim of providing an ecological snapshot of the variation in contributors to city-level life expectancy gaps and consistent with previous research using the Arriaga method,^9,12,16,26,27^ we did not calculate confidence intervals for cause of death contributions or statistically compare decomposed life expectancy gaps across cities. We conducted analyses using SAS statistical software version 9.4 (SAS Institute) and Excel software version 2013 (Microsoft).

## Results

### Population and Socioeconomic Characteristics of Selected Cities

The 3 cities are located in different regions of the US and differ across mortality, population, and socioeconomic characteristics (Table 1). Los Angeles is the most populous of the 3 cities, more than 6 times the size of Baltimore. The percentages of Black and White populations and age distributions vary across the cities. Compared with the US overall, all cities fare worse on selected socioeconomic characteristics. Baltimore had the highest levels of individual poverty, unemployment, neighborhood racial and ethnic segregation, and income inequality; Houston had the highest uninsured rate; and Los Angeles had the lowest high school completion rate.

### Study Population

We extracted a total of 95 776 death records for this analysis: 48 911 records from Los Angeles, 34 088 from Houston, and 12 777 from Baltimore. Of those records, we excluded 12 with unstated age and 29 443 where the decedent’s race and ethnicity was not non-Hispanic Black or non-Hispanic White. The final analysis included 66 321 records (29 134 from Los Angeles, 24 663 from Houston, and 12 524 from Baltimore). Of these, 29 057 individuals (44%) were identified as Black, 34 745 (52%) as male, and 46 128 (70%) as aged 65 years and older.

### Black to White Life Expectancy Gaps by City

Table 1 shows Black and White life expectancies and overall Black to White life expectancy gaps for Baltimore, Houston, and Los Angeles in 2018 and 2019. Los Angeles had the highest Black and White life expectancies overall (71.98 and 81.55 years, respectively), whereas Houston had the lowest (68.75 and 76.82 years, respectively). All 3 cities had Black to White life expectancy gaps of more than 7 years, with Los Angeles having the largest gap (9.57 years), Baltimore having the lowest gap (7.60 years), and Houston having a gap of 8.06 years.

Life expectancy calculations by sex revealed similar patterns across the 3 cities, along with some noticeable differences (eTable 2 in Supplement 1). Los Angeles had the highest Black and White life expectancies for both male and female individuals and the largest racial gap for male and female individuals (10.64-year and 8.65-year gaps, respectively). Houston had the lowest life expectancies for female individuals and White male individuals and the lowest male Black to White life expectancy gap (8.98 years). Baltimore had the lowest life expectancy for Black men, but also had the lowest female Black to White life expectancy gap (5.70 years).

### Cause-of-Death Contributions to Black to White Life Expectancy Gaps by City

We show the contributions of 26 causes of death to the Black to White life expectancy gap for each city in Table 2. Across cities, circulatory diseases, cancer, injuries, and diabetes and endocrine disorders were the top 4 contributors to the life expectancy gap, although the order and magnitude of these top contributors varied.

**Table 2.** Contributions of Specific Causes of Death to Life Expectancy Gaps between Non-Hispanic Black and Non-Hispanic White Populations in 3 Large US Cities, 2018-2019

Cause of death	Contribution to Black to White gap, No. of years (% contribution)		
	Los Angeles, California	Houston, Texas	Baltimore, Maryland
Total Black to White life expectancy gap, y	9.57	8.06	7.60
Circulatory diseases			
All	3.76 (39.3)	2.53 (31.4)	2.12 (28.0)
Heart disease	2.70 (28.2)	1.77 (21.9)	1.28 (16.9)
Stroke	0.54 (5.6)	0.48 (5.9)	0.53 (7.0)
Other circulatory diseases	0.53 (5.5)	0.29 (3.6)	0.31 (4.1)
Cancer			
All	1.44 (15.0)	1.30 (16.1)	1.17 (15.5)
Lung	0.24 (2.5)	0.22 (2.7)	0.20 (2.7)
Breast	0.15 (1.6)	0.18 (2.2)	0.11 (1.4)
Colorectal	0.18 (1.9)	0.15 (1.9)	0.12 (1.6)
All other	0.87 (9.1)	0.75 (9.3)	0.75 (9.8)
Injuries			
All	1.36 (14.2)	1.11 (13.8)	2.22 (29.3)
Suicide	0.02 (0.2)	−0.19 (−2.4)	−0.23 (−3.0)
Homicide	0.77 (8.1)	0.90 (11.1)	1.93 (25.4)
Accidents	0.64 (6.7)	0.50 (6.2)	0.22 (3.0)
Opioid overdose	−0.13 (−1.3)	−0.15 (−1.8)	0.30 (3.9)
Other unintentional injuries	0.05 (0.5)	0.06 (0.7)	0.01 (0.1)
Diabetes and endocrine disorders			
All	0.74 (7.8)	0.63 (7.8)	0.58 (7.7)
Diabetes	0.62 (6.5)	0.43 (5.4)	0.42 (5.6)
Other endocrine, metabolic, and glucose regulation disorders	0.12 (1.3)	0.20 (2.4)	0.16 (2.1)
Respiratory diseases			
All	0.62 (6.4)	0.30 (3.8)	0.06 (0.8)
Chronic lower respiratory disease	0.38 (4.0)	0.05 (0.6)	0.00 (0.0)
Influenza and pneumonia	0.19 (2.0)	0.14 (1.8)	0.04 (0.6)
Other respiratory diseases	0.04 (0.4)	0.11 (1.4)	0.01 (0.2)
Liver and kidney diseases			
All	0.40 (4.1)	0.33 (4.1)	0.12 (1.6)
Chronic liver disease and cirrhosis	0.07 (0.7)	−0.05 (−0.6)	−0.07 (−0.9)
Kidney disease	0.35 (3.5)	0.38 (4.7)	0.20 (2.6)
Perinatal conditions^a^	0.26 (2.7)	0.52 (6.5)	0.28 (3.7)
HIV and other infectious diseases	0.22 (2.3)	0.61 (7.6)	0.44 (5.8)
Nervous system disease	0.19 (2.0)	0.13 (1.7)	−0.01 (−0.1)
Mental or behavioral health disorders	0.13 (1.4)	0.06 (0.7)	0.21 (2.7)
Digestive system disease	0.09 (1.0)	0.13 (1.6)	0.10 (1.3)
Other diseases of the genitourinary system	0.05 (0.5)	0.04 (0.5)	0.02 (0.2)
All other causes	0.31 (3.3)	0.35 (4.4)	0.27 (3.5)

^a^ Includes congenital and chromosomal abnormalities and maternal deaths in the perinatal period that were coded as such on the death record.

In Los Angeles and Houston, circulatory diseases contributed the most to the Black to White life expectancy gap (3.76 years [39.3%] and 2.53 years [31.4%], respectively), followed by cancer, injuries, and diabetes and endocrine disorders. However, in Baltimore, injuries were the largest contributor, explaining 2.22 years (29.3%) in the gap, followed by circulatory diseases (2.12 years [28.0%]), cancer, and diabetes and endocrine disorders. Between Los Angeles and Baltimore, the contribution of circulatory diseases varied by 11.3 percentage points, with this difference primarily associated with a higher contribution of heart disease deaths in Los Angeles. Additionally, the contribution of injuries to the gap in Baltimore (2.22 years [29.3%]) was more than double its contribution in Los Angeles (1.36 years [14.2%]) and Houston (1.11 years [13.8%]). This difference was primarily associated with the contribution of homicide to Baltimore’s racial gap (1.93 years [25.4%]), which was more than double that in Houston (0.90 years [11.1%]) and triple that in Los Angeles (0.77 years [8.1%]).

We also noted several differences by city beyond the top contributing causes. Respiratory disease, which includes pneumonia and influenza, was a greater contributor to the life expectancy gap in Los Angeles (0.62 years [6.4%]) than in the other cities. Liver and kidney diseases contributed more to the gaps in Los Angeles (0.40 years [4.1%]) and Houston (0.33 years [4.1%]) than Baltimore (0.12 years [1.6%]). Perinatal conditions contributed almost double to Houston’s gap (0.52 years [6.5%]) compared with Los Angeles and Baltimore. The contribution of HIV and other infectious diseases in Houston (0.61 years [7.6%]) was more than triple that in Los Angeles (0.22 years [2.3%]). Finally, there appear to be differences in the contributions of accidents, which include some overdoses not classified as opioids, and opioid overdoses across the cities.

### Cause-of-Death Contributions to Sex-Specific Black to White Life Expectancy Gaps by City

**Figure 1.**
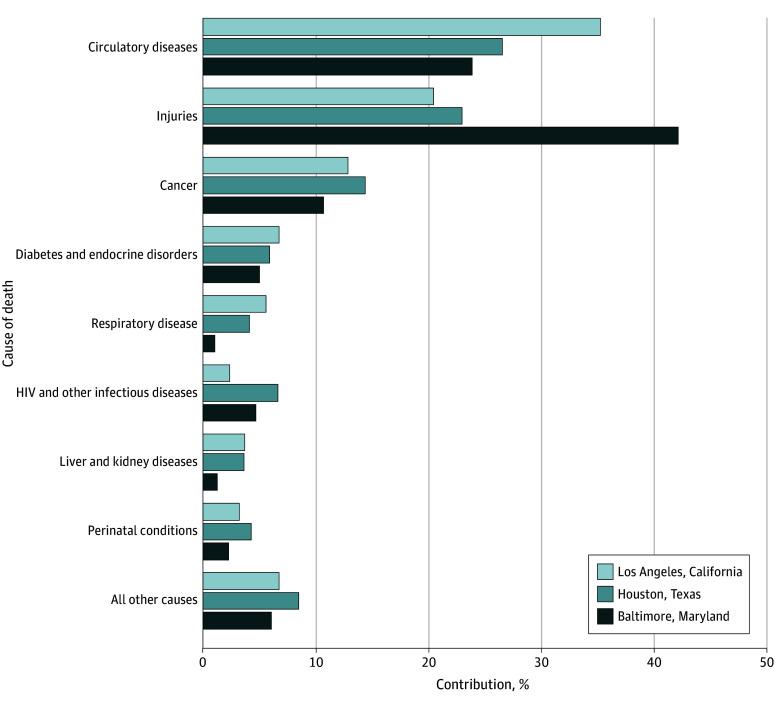
Percentage Contributions to the Black to White Life Expectancy Gap for Male Individuals in 3 Cities, 2018-2019 Figure only includes causes of death that accounted for 3% or more of the Black to White gap in at least 1 of the 3 cities. Male Black to White life expectancy gaps were 10.64 years (68.20 years vs 78.85 years) in Los Angeles, 8.98 years (64.97 years vs 73.95 years) in Houston, and 9.78 years (64.39 years vs 74.17 years) in Baltimore.

**Figure 2.**
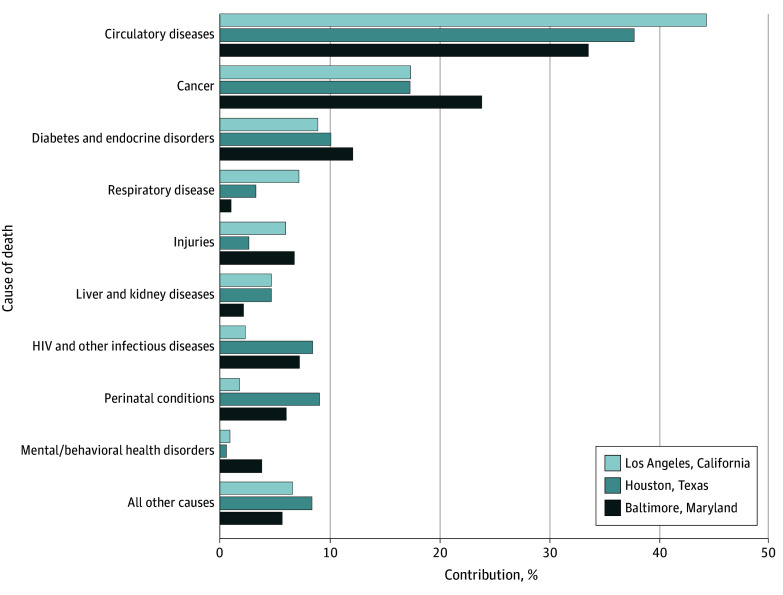
Percentage Contributions to the Black to White Life Expectancy Gap for Female Individuals in 3 Cities, 2018-2019 Figure only includes causes of death that accounted for 3% or more of the Black to White gap in at least 1 of the 3 cities. Female Black to White life expectancy gaps were 8.65 years (75.74 years vs 84.39 years) in Los Angeles, 7.42 years (72.39 years vs 79.81 years) in Houston, and 5.7 years (75.68 years vs 81.38 years) in Baltimore.

We assessed the ranking and magnitude of the contribution for causes of death that accounted for 3% or more of the Black to White life expectancy gap in at least 1 of the cities among male individuals (Figure 1) and female individuals (Figure 2). We included detailed results in eTable 2 in Supplement 1.

Among male individuals, circulatory diseases, injuries, and cancer were the top 3 contributors to the racial gap; however, as found in the overall analysis, injuries (namely, homicides) were the top contributor to the male Black to White life expectancy gap in Baltimore (4.25 years [43.4%]) but not in Los Angeles (2.24 years [21.1%]) and Houston (2.12 years [23.7%]). In addition, circulatory diseases played a larger role in the male racial gap in Los Angeles (3.87 years [36.3%]) than in Houston (2.46 years [27.4%]) and in Baltimore (2.41 years [24.6%]). We otherwise observed similar trends among male individuals across cities as discussed in our overall analysis.

Our findings among female individuals differed because of the lower magnitude of the contribution of injuries to the life expectancy gaps. In all 3 cities, circulatory diseases, cancer, and diabetes and endocrine disorders were the top 3 contributors to the female racial gaps, in this same order. As with the overall findings, circulatory diseases had a greater contribution in Los Angeles (3.75 years [43.4%]) than in Houston (2.74 years [37.0%]) and in Baltimore (1.87 years [32.9%]). However, cancer explained a noticeably greater proportion of the female Black to White life expectancy gap in Baltimore (1.33 years [23.4%]) than in Los Angeles (1.47 years [17.0%]) and Houston (1.26 years [16.9%]). Beyond the top 3 contributors, we observed variation in the contributors and their magnitudes across the 3 female life expectancy gaps (Figure 2).

Overall, comparing male with female individuals, circulatory diseases, cancer, and diabetes and endocrine disorders explained a meaningfully larger proportion of the female Black to White life expectancy gap than the male gap. Conversely, injuries (particularly homicide) played a larger role in the gaps for male individuals.

## Discussion

In this cross-sectional study of 2018 to 2019 mortality data from 3 large US cities, we found Black to White life expectancy gaps ranging from 7.60 to 9.57 years, primarily in association with the disproportionate burden of deaths due to chronic conditions (circulatory disease, cancer, and diabetes and endocrine disorders) and homicide among Black populations. The ordering and magnitude contributed by these causes of death to the racial life expectancy gap differed across cities and by sex, with injuries being the top contributor in Baltimore, particularly among men, and circulatory disease the top contributor in Los Angeles.

As an increasing number of US government agencies, health systems, and national organizations, such as the American Medical Association, have named racism a public health crisis,^28,29,30^ many jurisdictions are seeking evidence-informed strategies to respond. Substantial gaps between the life expectancies of Black and White individuals in major cities are a fundamental indicator of structural racism’s deleterious effects. Despite this, previous researchers have noted that “the mortality experience in the urban core of major U.S. cities has received little attention.”^26^ In particular, gaps in Black to White life expectancies and the causes of death associated with those inequities are understudied at the local level. The 3 geographically and demographically diverse cities within our analysis vary by population, proportion of Black residents, and socioeconomic characteristics, but all had racial life expectancy gaps of more than 7 years. Our analysis highlights the similarities and differences in contributors to the Black to White life expectancy gaps across selected cities and underlines how a deeper understanding of city-level mortality can support local efforts to improve racial health equity.

Our findings aligned in many ways with studies in other US cities and counties,^4,15,16^ with chronic diseases being top contributors overall and homicide being a top contributor among male individuals. Compared with an older study (with a different analytical strategy) of contributing causes in Los Angeles County, which includes the city of Los Angeles and surrounding areas, the relative impact of HIV has substantially decreased and circulatory diseases have become the leading contributor instead.^15^

Although several city-level studies have examined contributors to increases in overall life expectancy over time,^27,31^ few studies have focused on racial inequities and the factors associated with racial gaps. As Ho et al^15^ noted, this distinction is important because initiatives designed to improve overall health outcomes are different from those aiming to improve equity within them. Many cities, including Chicago and Baltimore, are now focused on addressing racial gaps in life expectancy as part of their strategic health plans.^4,32^ However, substantial improvements will be achieved only if cities understand underlying inequities and leverage funding to address these inequities.

Our data form a snapshot of 3 diverse US cities, highlighting the need for local health departments to have data specific to their jurisdictions and to consider how their demographic compositions and socioeconomic histories may shape the inequities experienced by Black and other populations that are often marginalized. For example, there was minimal variation in the contribution of cancer types by city, but chronic lower respiratory disease played a larger role in Los Angeles. Understanding these layers will help committed partners in designing initiatives to achieve life expectancy equity locally. In particular, understanding local variability in mortality gaps (and other health outcomes) can guide more effective resource allocations and program planning.^33^

Reducing life expectancy inequities requires a multisector and intersectional approach that addresses root causes like structural racism and other “-isms,” such as classism and sexism, economic policies, and social factors (eg, food security), as well as downstream factors such as the disproportionate burden of respiratory disease, stress, and high blood pressure in Black populations.^34,35^ Our analysis accentuates the importance of guiding action to address root causes and downstream factors with local data. Although underlying root causes of inequities are similar across cities, our findings show that they likely work through different mechanisms and interact in unique ways within local contexts to produce variations in the top contributors to city-specific Black to White life expectancy gaps. An exploration of the reasons why there is variation between cities is beyond the scope of this article; however, our results show a need for cities to seek deeper understanding of the ways root causes affect pathways to well-being in their own localities.

By tying multisectoral work to life expectancy inequities and the most influential causes, cities can rally a diverse group around a common goal. Using life expectancy as a uniting goal for equity provides an evidence-informed approach, as well as a common language and narrative for advocates to highlight injustice. Life expectancy is a simple and powerful measure to understand and to communicate more broadly; moving beyond overall life expectancy to identify the contributing causes of life expectancy gaps creates more tangible information that can be used to guide action. Cross-sector partners can more easily see the ways their work ties to a shared goal, thus motivating further action and collaboration. In particular, our work highlights how the actions of medical practitioners relate to public health, connecting them to initiatives that address the root causes of well-being and fostering a greater alliance between medicine, public health, and other committed partners.

### Limitations

This study has limitations that should be addressed. We focused on Black to White inequities because the Black population in the US has historically experienced the poorest mortality outcomes of groups large enough to analyze in this manner. Native peoples also experience unjust mortality outcomes^36^ that require deeper understanding, particularly because this group is often overlooked by methods^37^ that are not chosen or driven by Indigenous peoples and nations.^38^ We recommend that jurisdictions examine life expectancy gaps and contributors for other racial and ethnic groups to understand their unique inequities, particularly because some have seen decreases in life expectancy over time even before the COVID-19 pandemic.^39^ Our analyses of the Black to White life expectancy gap among female and male individuals are based on the sex indicated on death records, not self-reported gender; therefore, our analysis does not identify the contributors to the Black to White life expectancy gap for transgender and nonbinary or gender-fluid populations, which likely have different top contributors.

We conducted analyses using data from before the COVID-19 pandemic, which has increased racial life expectancy gaps across the US and in many cities.^40,41,42^ The population denominator data used for this analysis comes from the 2010 US Census, which is the most recent source available with the age-specific population counts required to calculate life expectancy. Disproportionate changes in these cities’ demographics between 2010 and the study period may bias our results. Because this analysis categorized deaths into their respective underlying causes using *ICD-10* codes, we were unable to distill the contribution of infant mortality as defined as the death of a child younger than 1 year; however, the *ICD-10* code for perinatal conditions likely captures a meaningful proportion of infant deaths. In addition, researchers have noted that our approach does not distinguish the role of premature death from the role of different causes of death in producing the Black to White life expectancy gap.^43^ This is particularly true for chronic diseases, for which past gains in the Black to White life expectancy gap were due to narrowing gaps in the age of death from chronic diseases, not reduced likelihood of death from chronic diseases among Black populations.^43^ Furthermore, per Geronimus et al,^44^ we may overestimate the impact of specific causes of death because the method used overlooks competing causes of death (by assuming competing risks are independent). This would be particularly true for deaths among younger people, such as those related to infant mortality, homicide, and injuries.^44^

## Conclusions

Life expectancy is one of the paramount measures of health, and our decomposition of the racial life expectancy gap by cause of death provides valuable information regarding avenues for improving health equity at the local level. By assessing the composition of Black to White life expectancy gaps across 3 large US cities and categorizing deaths at a more granular level than past studies, we provide insight into differing contributors to urban inequities. This type of data (local, specific to cause of death, and comparative) can help committed partners (eg, local policy makers, health departments, community organizations and groups, funders, and advocacy partners) allocate resources more effectively to improve racial equity in our largest urban areas.

## Notes

Supplement 1. **eTable 1.** *International Classification of Diseases, 10th Edition (ICD-10) *Classified Into 26 Categories and 13 Classifications **eAppendix.** Life Expectancy Decomposition Formula
eReference
**eTable 2.** Sex-Specific Contributions of Specific Causes of Death to Life Expectancy Gap Between Non-Hispanic Black and Non-Hispanic White Individuals in Three Large US Cities

Supplement 2.
Data Sharing Statement
