# Supplementary material for: Life Expectancy Gaps Among Black and White Persons and Contributing Causes of Death in 3 Large US Cities, 2018-2019
Source: JAMA Netw Open. 2023 Mar 10;6(3):e233146. doi: 10.1001/jamanetworkopen.2023.3146 (PMC12549101; doi:10.1001/jamanetworkopen.2023.3146)
Supplement: Supplement 1. — eTable 1. International Classification of Diseases, 10th Edition (ICD-10) Classified Into 26 Categories and 13 Classifications eAppendix. Life Expectancy Decomposition Formula eReference eTable 2. Sex-Specific Contributions of Specific Causes of Death to Life Expectancy Gap Between Non-Hispanic Black and Non-Hispanic White Individuals in Three Large US Cities [file jamanetwopen-e233146-s001.pdf]

Supplemental Online Content

Roesch PT, Saiyed NS, Laflamme E, De Maio FG, Benjamins MR. Life expectancy gaps among Black and White persons and contributing causes of death in 3 large US cities, 2018-2019. *JAMA Netw Open*. 2023;6(3):e233146. doi:10.1001/jamanetworkopen.2023.3146

eTable 1. *International Classification of Diseases, 10th Edition (ICD-10)* Classified Into 26 Categories and 13 Classifications

eAppendix. Life Expectancy Decomposition Formula

eReference

eTable 2. Sex-Specific Contributions of Specific Causes of Death to Life Expectancy Gap Between Non-Hispanic Black and Non-Hispanic White Individuals in Three Large US Cities

This supplemental material has been provided by the authors to give readers additional information about their work.

© 2023 Roesch PT et al. *JAMA Network Open.*

eTable 1. ***International Classification of Diseases, 10th Edition (ICD-10)*** **Classified Into 26 Categories and 13 Classifications**

Cause	ICD-10 Codes
**Circulatory Diseases**	
Heart disease	I0, I11, I13, I2-I4, I50, I51
Stroke	I6
Other circulatory diseases	I7-I10, I12
**Cancer**	
Lung cancer	C33, C34
Breast cancer	C50
Colorectal Cancer	C18-C20, C26
All other cancers	C0, C4, C6-C17, C21-C25, C30-C32, C37-C39, C51-C58, D0-D3, D40-D48
**Diabetes and Endocrine Disorders**	
Diabetes mellitus	E10-E14
Other Endocrine/Metabolic/Glucose Regulation	E0, E2-E9, E15-E19
**Respiratory Disease**	
Chronic lower respiratory disease	J4
Influenza and pneumonia	J1, J09
Other respiratory diseases	J2, J3, J5-J9
**Liver and Kidney Diseases**	
Chronic liver disease and cirrhosis	K70, K73, K74
Kidney Disease	N00-N07, N17-N19, N25-N27
**Perinatal conditions, including Congenital and Chromosomal Abnormalities**	P, O, Q
**Injuries**	
Suicide	X6, X7, X80-X84, Y870
Homicide	X85-X89, X9, Y0, Y871
Accidents	V, W, X0-X5, Y85, Y86
Opioid Overdose	X40-X44, X60-X64, X85, Y10-Y14 + Contributing Cause T40.0-T40.4, T40.6
Other unintentional injuries	Y1-Y7, Y9, Y80-Y84, Y88
**HIV and Other Infectious Diseases**	A0-A9, B0, B1, B20-B29, B3-B9
**Mental/Behavioral health disorders**	F
**Nervous System Disease**	G
**Digestive System Disease**	K0-K6, K8, K9, K71, K72, K75-K77
**Other Diseases of the genitourinary system**	N20-N23, N10-N13, N15, N28, N30-N40, N70-N76, N4, N6, N8, N9
**All Other Causes**	H, L, M, R, D5-D8

© 2023 Roesch PT et al. *JAMA Network Open.*

eAppendix. Life Expectancy Decomposition Formula[1]

$$ {}_n\Delta_x^i = {}_n\Delta_x * \frac{{}_nm_x^i(2) - {}_nm_x^i\,(1)}{{}_nm_x(2)\,{}_nm_x\,(1)};\ {}_n\Delta_x = \frac{{}_nR_x^i(2) * {}_nm_x(2) - {}_nR_x^i(1) * {}_nm_x\,(1)}{{}_nm_x(2) - {}_nm_x\,(1)} $$

Where:

${}_nm_x^i(2), {}_nm_x^i\,(1) =$ specific contribution of differences in mortality rates from cause i between ages x and x + n in populations 1 and 2.

$nR_x^i(1)(2) =$ the proportion of deaths from cause i between ages x and x + n in population 1 and 2.

$n\Delta_x^i$= contribution of all-cause mortality differences in age group x to x+n to differences in e_0^0

eReference:

1. Preston S, Heuveline P, Guillot M. In Demography: Measuring and Modeling Population Processes In: *Multiple Decrement Processes.* Blackwell Publishers; 2002:71–97.

© 2023 Roesch PT et al. *JAMA Network Open.*

eTable 2. Sex-Specific Contributions of Specific Causes of Death to Life Expectancy Gap Between Non-Hispanic Black and Non-Hispanic White Individuals in Three Large US Cities

	Baltimore				Houston				Los Angeles			
	Males		Females		Males		Females		Males		Females	
**NH Black Life Expectancy (in years)**	64.39		75.68		64.97		72.39		68.20		75.74	
**NH White Life Expectancy (in years)**	74.17		81.38		73.95		79.81		78.85		84.39	
**Total Life Expectancy Gap (in years)**	9.78		5.70		8.98		7.42		10.64		8.65	
	**Contribution to Black to White Gap**				**Contribution to Black to White Gap**				**Contribution to Black to White Gap**			
**Causes of Death**	**Years**	**Percent**	**Years**	**Percent**	**Years**	**Percent**	**Years**	**Percent**	**Years**	**Percent**	**Years**	**Percent**
**Circulatory Diseases**	2.41	24.6%	1.87	32.9%	2.46	27.4%	2.74	37.0%	3.87	36.3%	3.75	43.4%
Heart disease	1.65	16.9%	0.96	16.8%	1.77	19.8%	1.91	25.7%	2.97	27.9%	2.49	28.8%
Stroke	0.41	4.2%	0.66	11.5%	0.43	4.8%	0.51	6.9%	0.44	4.1%	0.66	7.6%
Other Circulatory Diseases	0.35	3.5%	0.26	4.6%	0.25	2.8%	0.33	4.4%	0.46	4.3%	0.61	7.0%
**Cancer**	1.08	11.0%	1.33	23.4%	1.33	14.8%	1.26	16.9%	1.41	13.3%	1.47	17.0%
Lung Cancer	0.26	2.7%	0.14	2.4%	0.31	3.5%	0.11	1.4%	0.27	2.5%	0.21	2.5%
Breast Cancer	0.00	0.0%	0.19	3.3%	0.01	0.1%	0.32	4.4%	0.00	0.0%	0.28	3.2%
Colorectal Cancer	0.10	1.0%	0.15	2.6%	0.16	1.8%	0.15	2.0%	0.15	1.4%	0.21	2.5%
All Other Cancers	0.72	7.3%	0.86	15.1%	0.84	9.4%	0.68	9.1%	0.99	9.3%	0.77	8.9%
**Diabetes and Endocrine Disorders**	0.50	5.2%	0.68	11.9%	0.55	6.1%	0.74	9.9%	0.74	7.0%	0.76	8.7%
Diabetes Mellitus	0.37	3.7%	0.49	8.7%	0.43	4.8%	0.44	5.9%	0.62	5.8%	0.63	7.3%
Other Endocrine/Metabolic/Glucose Regulation	0.14	1.4%	0.18	3.2%	0.11	1.3%	0.30	4.0%	0.13	1.2%	0.12	1.4%
**Respiratory Disease**	0.10	1.0%	0.06	1.0%	0.38	4.2%	0.23	3.1%	0.62	5.8%	0.61	7.1%
Chronic Lower Respiratory Disease	0.00	0.0%	0.02	0.3%	0.09	1.0%	0.00	-0.1%	0.38	3.6%	0.37	4.3%
Influenza and Pneumonia	0.05	0.5%	0.04	0.7%	0.13	1.5%	0.16	2.1%	0.18	1.7%	0.22	2.5%

© 2023 Roesch PT et al. *JAMA Network Open.*

Other Respiratory Diseases	0.05	0.5%	0.00	0.0%	0.16	1.8%	0.07	1.0%	0.06	0.5%	0.02	0.2%
**Liver and Kidney Diseases**	0.13	1.3%	0.12	2.0%	0.34	3.8%	0.34	4.6%	0.41	3.8%	0.40	4.6%
Chronic Liver Disease and Cirrhosis	-0.03	-0.3%	-0.11	-1.9%	-0.04	-0.4%	-0.04	-0.6%	0.08	0.8%	0.06	0.7%
Kidney Disease	0.16	1.6%	0.22	3.9%	0.37	4.2%	0.38	5.2%	0.32	3.0%	0.34	3.9%
**Perinatal Conditions[a]**	0.23	2.3%	0.34	6.0%	0.40	4.4%	0.66	8.9%	0.36	3.3%	0.15	1.7%
**Injuries**	4.25	43.4 %	0.38	6.6%	2.12	23.7 %	0.18	2.5%	2.24	21.1 %	0.51	5.9%
Suicide	-0.33	-3.4%	-0.05	-0.9%	-0.20	-2.2%	-0.12	-1.6%	0.10	1.0%	-0.03	-0.4%
Homicide	3.53	36.1 %	0.29	5.1%	1.54	17.2 %	0.23	3.1%	1.27	11.9 %	0.25	2.9%
Accidents	0.34	3.5%	0.15	2.7%	0.92	10.3 %	0.09	1.2%	0.97	9.1%	0.32	3.7%
Opioid Overdose	0.68	6.9%	0.00	0.0%	-0.23	-2.5%	-0.04	-0.5%	-0.18	-1.7%	-0.04	-0.4%
Other Unintentional Injuries	0.04	0.4%	-0.01	-0.3%	0.08	0.9%	0.03	0.4%	0.08	0.8%	0.01	0.1%
**HIV and Other Infectious Diseases**	0.48	4.9%	0.41	7.1%	0.62	6.9%	0.61	8.3%	0.26	2.4%	0.19	2.2%
**Mental/Behavioral Health Disorders**	0.19	2.0%	0.21	3.6%	0.07	0.8%	0.04	0.6%	0.18	1.7%	0.08	0.9%
**Nervous System Disease**	0.07	0.7%	-0.08	-1.4%	0.14	1.5%	0.14	1.9%	0.20	1.9%	0.18	2.0%
**Digestive System Disease**	0.09	1.0%	0.12	2.1%	0.16	1.7%	0.11	1.5%	0.07	0.6%	0.12	1.4%
**Other Diseases of the Genitourinary System**	0.02	0.2%	0.02	0.4%	0.04	0.4%	0.04	0.6%	0.04	0.4%	0.06	0.7%
**All Other Causes**	0.23	2.4%	0.25	4.4%	0.38	4.3%	0.32	4.4%	0.25	2.4%	0.38	4.4%

Notes: [a] Includes Congenital and Chromosomal Abnormalities
NH = non-Hispanic

© 2023 Roesch PT et al. *JAMA Network Open.*
