# Supplementary material for: Life Expectancy Gaps Among Black and White Persons and Contributing Causes of Death in 3 Large US Cities, 2018-2019
Source: JAMA Netw Open. 2023 Mar 10;6(3):e233146. doi: 10.1001/jamanetworkopen.2023.3146 (PMC12549101; doi:10.1001/jamanetworkopen.2023.3146)
Supplement: Supplement 2. — Data Sharing Statement [file jamanetwopen-e233146-s002.pdf]

Data Sharing Statement

Roesch. Life Expectancy Gaps Among Black and White Persons and Contributing Causes of Death in 3 Large US Cities, 2018-2019. *JAMA Netw Open*. Published March 10, 2023. doi:10.1001/jamanetworkopen.2023.3146

Data
Data available: No

Additional Information
Explanation for why data not available: Our DUA with NCHS does not allow the sharing of this Restricted Use data set.
